# Performance of DeepSeek V3 and ChatGPT-4o in answering esophageal cancer-related questions

**DOI:** 10.1097/MD.0000000000049896

**Published:** 2026-07-24

**Authors:** Qian Yang, Jing Yi, Anran Gong, Yanyan Li, Qingyan Feng, Aiyan Fu, Jiangtao Li, Yunkai Zhan

**Affiliations:** a Department of Gastroenterology, Jiujiang City Key Laboratory of Cell Therapy, Jiujiang No. 1 People’s Hospital, Jiujiang, Jiangxi, China; b Department of Clinical Medicine, Medical College, Jinzhou Medical University, Jinzhou, Liaoning, China.

**Keywords:** ChatGPT-4o, DeepSeek V3, esophageal cancer, health knowledge

## Abstract

Esophageal cancer remains a significant global health issue. ChatGPT-4o and DeepSeek V3 can provide the public with health-related knowledge about esophageal cancer. This study aimed to evaluate the accuracy of DeepSeek V3 and ChatGPT-4o in responding to health knowledge questions related to esophageal cancer. Fifty-two questions related to esophageal cancer were classified into themes of basic knowledge, diagnosis and molecular biology, management of local and locoregional diseases, management of advanced and metastatic diseases, clinical case analysis and patient frequently asked questions (FAQs). These questions were entered into DeepSeek V3 and ChatGPT-4o to obtain responses, and 2 experienced gastroenterologists independently evaluated the accuracy and temporal stability of each response. Overall, the scores of DeepSeek V3 and ChatGPT-4o on all questions were 4 (3–4), and there was no statistically significant difference between the 2 groups. The final scores of DeepSeek V3 in basic knowledge, diagnosis and molecular biology, management of local and locoregional diseases, management of advanced and metastatic diseases, clinical case analysis, and FAQs were 4 (3–4), 4 (3–4), 4 (3–4), 3 (3–4), 4 (4–4), and 4 (4–4), respectively, while the scores of ChatGPT-4o were 4 (3–4), 3 (2–4), 4 (3–4), 3 (3–4), 4 (4–4), and 4 (4–4), respectively. For temporal stability across 2 independent test runs, DeepSeek V3 presented inconsistent responses on 2 questions, and ChatGPT-4o on 1 question; no statistically significant differences were found in overall and subgroup scores between the 2 runs for both models (all *P* > .05). ChatGPT-4o and DeepSeek V3 showed favorable accuracy and comprehensive responses to most of the 52 esophageal cancer-related questions in this study, but our findings do not confirm their general reliability for esophageal cancer health information in routine clinical or public use.

## 1. Introduction

Esophageal cancer is a malignant tumor with the eleventh highest incidence rate and the seventh leading cause of cancer mortality in the world.^[1]^ Esophageal cancer remains a significant global health issue, mainly with 2 subtypes: esophageal squamous cell carcinoma and esophageal adenocarcinoma.^[2]^ The main risk factors for esophageal adenocarcinoma are gastroesophageal reflux disease, abdominal obesity, and smoking, while most cases of esophageal squamous cell carcinoma are caused by alcohol consumption and smoking.^[3-5]^ As most patients are diagnosed with advanced diseases, the overall 5-year survival rate of esophageal cancer is still <20%.^[6]^ The screening methods for esophageal cancer mainly include white light endoscopy, barium meal angiography, capsule endoscopy, etc.^[7]^ A mixed-methods systematic review assessing public acceptance of screening strategies for esophageal cancer showed that intended participation in esophageal adenocarcinoma screening in questionnaire-based studies ranged from 62·8% to 71·4%.^[8]^ However, a population-based cohort study found that the public’s awareness of the symptoms of esophageal cancer was disconcertingly low.^[9]^ Raising public awareness and attention to esophageal cancer may help reduce the incidence and mortality of esophageal cancer.^[10]^

The rapid development of artificial intelligence (AI) profoundly affects people’s daily life. AI-enabled chatbots are increasingly being used in healthcare.^[11]^ Chat Generative Pre-trained Transformer (ChatGPT), one of the large language models (LLMs) developed by OpenAI, can create text by collecting information from various data sources and answering related questions.^[12]^ A study found that ChatGPT-4 performed better than residents in answering colorectal cancer-related questions but still needs more improvement.^[13]^ DeepSeek is a China-based AI model and is freely accessible to the public.^[14]^ This model has been significantly optimized in complex logical reasoning, mathematical calculation, and long document parsing, and can provide users with high-quality information integration and creative output. A recent study found that the DeepSeek large language model performs well in medical tasks and clinical reasoning, indicating that DeepSeek has potential value in medical applications.^[15]^ Sarah Sandmann et al included 125 patient cases to evaluate the performance of the DeepSeek model in clinical decision-making; the results showed that the performance of the DeepSeek model was similar to that of ChatGPT-4o.^[16]^ Recently, several studies have explored the utility of LLMs in esophageal cancer-related information services. In 2025, the European Society for Medical Oncology (ESMO) issued the ESMO Guidance on the Use of Large Language Models in Clinical Practice (ELCAP), the first specialized framework for safe and normative LLM application in oncology, which explicitly stresses that all LLM outputs in cancer care must be used under professional supervision and rigorous clinical validation to prevent misleading information and ensure patient safety.^[17]^ This authoritative guidance provides an essential normative basis for assessing the reliability of LLMs in esophageal cancer-related knowledge services. He et al conducted a multi-model assessment of LLMs for esophageal cancer question answering^[18]^; Cheng et al evaluated ChatGPT-4o-provided esophageal cancer information using the SERVQUAL framework^[19]^; and Yu et al assessed ChatGPT-4o’s performance in addressing patient-centered concerns about esophageal cancer.^[20]^ In the broader field of oncology, Rydzewski et al performed a large-scale comparative evaluation of LLMs across oncological topics.^[21]^ However, existing studies have primarily focused on single-model assessments or broad multi-model comparisons without a head-to-head evaluation of DeepSeek V3 and ChatGPT-4o – the latter being a latest-generation model and the former a representative Chinese open-access LLM. Furthermore, few studies have integrated ESMO guideline-based professional questions, patient frequently asked questions (FAQs), and clinical case analyses while simultaneously evaluating both accuracy and temporal stability. While our study was initially designed to focus on public-oriented esophageal cancer health knowledge, we recognized that large language models are also increasingly accessed by clinicians for rapid reference to clinical guidelines and case reasoning. To achieve a more holistic assessment, we expanded the question set to include not only public common questions but also technical diagnosis and management questions derived from the 2022 ESMO Clinical Practice Guidelines and 3 representative clinical cases. Accordingly, this study designed 52 standardized esophageal cancer-related questions to directly compare the performance of DeepSeek V3 and ChatGPT-4o, with the aim of clarifying their strengths and limitations and providing evidence for their clinical and public application.

## 2. Materials and methods

### 2.1. Data source

To evaluate the accuracy of DeepSeek V3 and ChatGPT-4o in answering esophageal cancer-related knowledge, we first organized and designed 13 questions on the most common basic knowledge of esophageal cancer. The 20 FAQs were systematically collected from outpatient/inpatient consultations in the Department of Gastroenterology, Jiujiang No. 1 People’s Hospital, standardized esophageal cancer patient education materials, and public online medical inquiry platforms. These were subsequently reviewed and confirmed by 2 senior gastroenterologists to ensure clinical representativeness and content validity. At the same time, the clinical practice guidelines for the diagnosis, treatment, and follow-up of esophageal cancer formulated by the ESMO in 2022 were selected as the source of the professional knowledge section of esophageal cancer. Recommendations were extracted from the guidelines, and statements of low or very low-quality evidence and weak recommendations were excluded. Subsequently, the extracted recommendations were modified into questions. For example, one of the guideline recommendations in the “second and subsequent lines of treatment for advanced esophageal SCC” section is “chemotherapy (ChT) with a taxane or irinotecan can be considered in fit patients who have been previously treated with platinum-fluoropyrimidine and/or nivolumab or pembrolizumab.” We would rephrase the above statement as “For suitable patients who have previously received with platinum-fluoropyrimidine and/or nivolumab or pembrolizumab treatment, which regimens are recommended as second-line treatment?,” and then entered into DeepSeek V3 and ChatGPT-4o for responses, respectively. In addition, to further evaluate the model’s ability in clinical inference, we developed 3 representative cases of esophageal cancer. As this study did not involve human participants, the Medical Ethics Committee of Jiujiang No. 1 People’s Hospital waived the need for ethical approval.

### 2.2. Inquiries and response generation

In this study, we used 2 LLMs that are freely accessible to the public: DeepSeek V3(https://www.deepseek.com) and ChatGPT-4o (https://chat.openai.com). DeepSeek V3 is a China-based AI model that was launched in December 2024, and is capable of performing tasks such as question answering, authoring, and code generation.^[14]^ ChatGPT-4o was launched in May 2024, and its database has been updated to 2023. ChatGPT-4o is capable of processing and generating various inputs and outputs. It has a significant improvement in processing speed, reduced latency, and has been enhanced in both text and code processing.^[22]^ Each question was entered independently using the “New chat” function, and its responses were subsequently recorded. Consistent with previous studies, 2 independent tests were performed on April 11, 2025 and April 18, 2025. Given that publicly accessible LLMs may undergo unannounced backend updates or parameter adjustments over time, these 2 runs were designed to evaluate the temporal stability of model responses, rather than strict technical temporal stability under fully fixed conditions.^[23,24]^ In addition, we disabled the memory and browsing history functions during the search process.^[25,26]^

### 2.3. Prompt standardization

To maximize temporal stability, each question was entered verbatim as an independent prompt without any additional instructions, prompt engineering, contextual information, or follow-up dialogue (Table S1, Supplemental Digital Content 1). Every question was submitted in a new chat session using the default public web interface of each model. No paraphrasing, formatting modifications, or manual editing of either the input questions or model responses was performed. Because the publicly accessible versions of DeepSeek V3 and ChatGPT-4o do not allow users to modify inference parameters (e.g., temperature, top-p, maximum output length, or system prompts), all responses were generated using the default model settings provided by the respective platforms. Memory and browsing history functions remained disabled throughout the study.

### 2.4. Grading system

Prior to the review, 2 experienced gastroenterologists reviewed the 2022 ESMO esophageal cancer guidelines to establish standardized evaluation criteria. The accuracy, completeness, and clinical risk of each response were assessed using a 4-point scoring system with explicit anchors; the 3 dimensions were integrated into a single total score with clear grade definitions^[27]^:

Comprehensive (4 points): 100% factually accurate, fully complete with all key clinical details; no clinical risk or misleading information.

Correct but not sufficient (3 points): 100% factually accurate; but incomplete (missing 1–2 minor clinical details); no erroneous or high-risk content.

Mixing correct and incorrect/outdated data (2 points): Contains both correct and factually incorrect/outdated content; may lead to inappropriate clinical judgment; moderate risk of misguidance.

Completely incorrect (1 point): Entirely factually wrong; directly contradicts guideline recommendations; high risk of serious clinical misguidance.

Reviewers also assessed the temporal stability of the 2 LLMs for 2 responses to each question. If the responses were similar between the 2 runs, only the first response was scored. If the 2 responses are different, they should be scored separately. For responses that were disputed by the 2 reviewers, the responses were reassessed by another senior gastroenterologist in a blinded manner to obtain the final score. The third senior gastroenterologist adjudicated only those responses for which the 2 initial reviewers disagreed; no independent rescoring of all items was performed.

### 2.5. Statistical analysis

Statistical analyses were performed using SPSS 26.0 and R 4.2.0. Between-model comparisons used the Wilcoxon signed-rank test, with effect size *r* = *Z*/√N (interpreted as ≥ 0.5 large, ≥0.3 moderate). Bootstrap 95% confidence intervals (1000 replicates) were calculated for mean score differences. Given small subgroup sample sizes (e.g., n = 3, n = 5), *P*-values are unstable; therefore, interpretation focuses on effect sizes and CIs. For multiple subgroup comparisons, Bonferroni correction was applied (corrected α = 0.05/*k*, where *k* = number of subgroups). Weighted kappa measured inter-rater reliability.^[28]^ All analyses were performed using SPSS version 26.0 (IBM, Armonk) and R version 4.2.0 (R Foundation for Statistical Computing, Vienna, Austria) for bootstrap and effect size calculations.

## 3. Results

### 3.1. Performance of DeepSeek V3 and ChatGPT-4o on esophageal cancer-related questions

**Figure 1.**
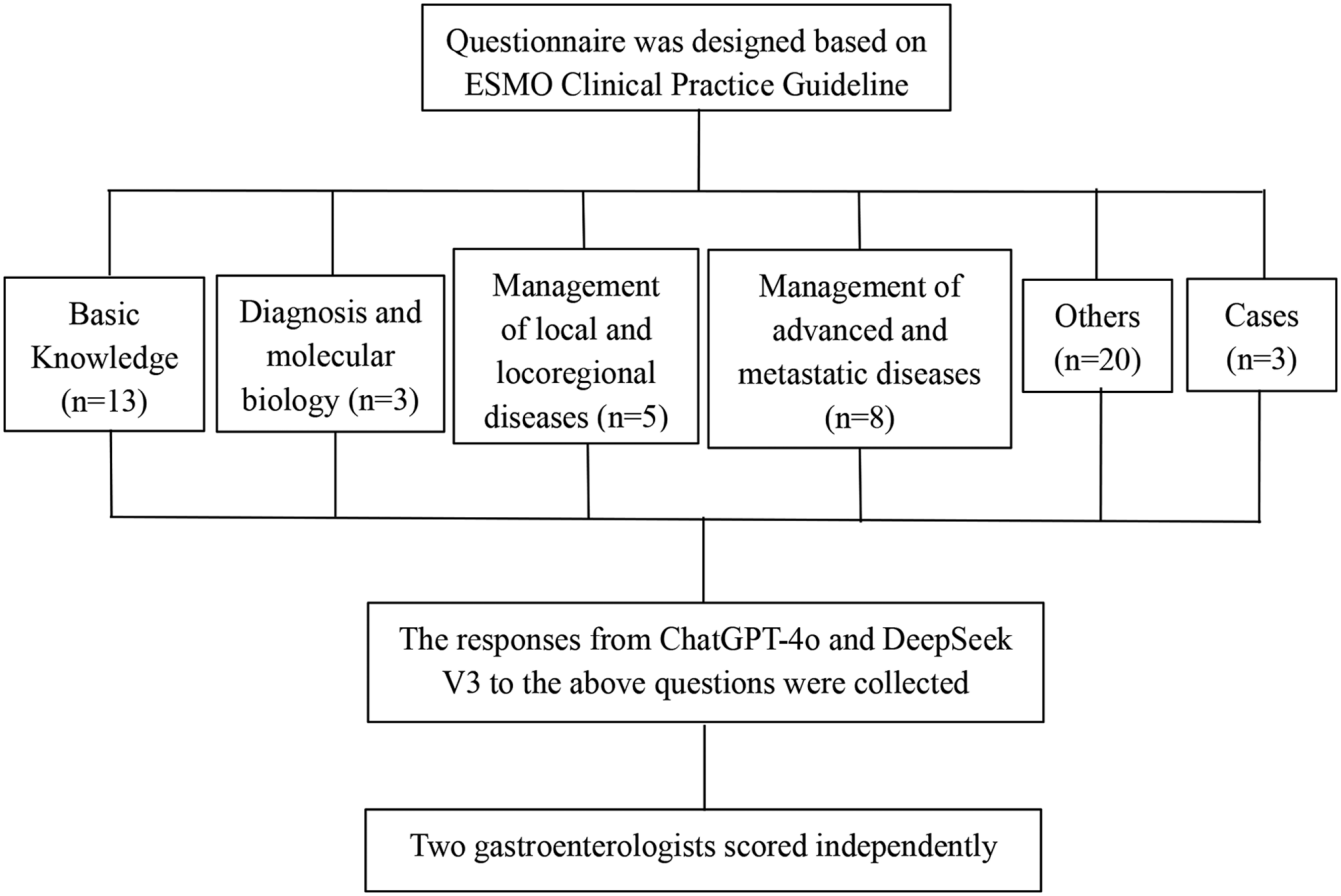
The flowchart of this study. ChatGPT = Chat Generative Pre-trained Transformer, ESMO = European Society of Medical Oncology.

Figure 1 illustrated the research flow of this study. A total of 52 esophageal cancer-related questions were included in this study, including 13 basic knowledge questions, 16 questions designed according to the ESMO Clinical Practice Guidelines for esophageal cancer, 20 frequently asked questions by patients, and 3 representative cases of esophageal cancer. The questions were classified into 6 categories based on their nature and source: basic knowledge (n = 13), diagnosis and molecular biology (n = 3), management of local and locoregional diseases (n = 5), management of advanced and metastatic diseases (n = 8), FAQs (n = 20), and clinical case analysis (n = 3). The FAQs were collected from outpatient/inpatient consultations and public online platforms, while the 3 case analyses were designed to evaluate clinical reasoning ability. Two gastroenterologists evaluated the responses from DeepSeek V3 and ChatGPT-4o, respectively, and the final score was determined by a third reviewer (Table S2, Supplemental Digital Content 2). Table 1 showed the performance of DeepSeek V3 and ChatGPT-4o on esophageal cancer-related questions. The final scores of DeepSeek V3 and ChatGPT-4o on all questions were 4 (3–4) and 4 (3–4), respectively, and there was no statistically significant difference between the 2 groups, yielding weighted kappa coefficients of 0.636 and 0.627, which represent moderate inter-rater agreement according to conventional statistical benchmarks. The inter-rater agreement reached only a moderate level, which reflects inevitable subjective judgment variability in manual scoring of LLM responses. This suggests that multicenter multidisciplinary expert panels are needed in future studies to further improve scoring consistency and objectivity. Domain-specific final scores for DeepSeek V3 were: basic knowledge 4 (3–4), diagnosis and molecular biology 4 (3–4), management of local and locoregional diseases 4 (3–4), management of advanced and metastatic diseases 3 (3–4), clinical case analysis 4 (4–4), and FAQs 4 (4–4). For ChatGPT-4o, the scores were 4 (3–4), 3 (2–4), 4 (3–4), 3 (3–4), 4 (4–4), and 4 (4–4), respectively.

**Table 1:** Performance of DeepSeek and ChatGPT 4o on esophageal cancer related questions.

	DeepSeek V3	ChatGPT-4o	Effect size	95% CI	*P* value
Overall					
Reviewer 1, median (IQR)	4 (3–4)	4 (3–4)	0.039	(−0.345, 0.424)	.938
Reviewer 2, median (IQR)	4 (4–4)	4 (4–4)	0.116	(−0.269, 0.500)	.510
Final scores, median (IQR)	4 (3–4)	4 (3–4)	0.139	(−0.246, 0.524)	.597
Basic knowledge					
Reviewer 1, median (IQR)	4 (3–4)	4 (3–4)	0.000	(−0.548, 0.548)	1.000
Reviewer 2, median (IQR)	4 (3–4)	4 (3–4)	0.000	(−0.548, 0.548)	.829
Final scores, median (IQR)	4 (3–4)	4 (3–4)	0.000	(−0.548, 0.548)	1.000
Diagnosis and molecular biology					
Reviewer 1, median (IQR)	4 (3–4)	3 (2–4)	0.816	(−1.544, 3.176)	.480
Reviewer 2, median (IQR)	3 (3–4)	4 (2–4)	0.000	(−2.266, 2.266)	1.000
Final scores, median (IQR)	4 (3–4)	3 (2–4)	0.816	(−1.544, 3.176)	.480
Management of local and locoregional diseases					
Reviewer 1, median (IQR)	4 (3–4)	4 (3–4)	0.270	(−1.194, 1.734)	.905
Reviewer 2, median (IQR)	4 (4–4)	3 (3–4)	0.895	(−0.634, 2.424)	.232
Final scores, median (IQR)	4 (3–4)	4 (3–4)	0.270	(−1.194, 1.734)	.905
Management of advanced and metastatic diseases					
Reviewer 1, median (IQR)	3 (3–3)	4 (3–4)	−0.500	(−1.590, 0.590)	.349
Reviewer 2, median (IQR)	4 (3–4)	4 (3–4)	0.153	(−0.922, 1.228)	.864
Final scores, median (IQR)	3 (3–4)	3 (3–4)	0.202	(−0.873, 1.277)	.811
FAQs					
Reviewer 1, median (IQR)	4 (4–4)	4 (4–4)	0.000	(−0.640, 0.640)	1.000
Reviewer 2, median (IQR)	4 (4–4)	4 (4–4)	0.000	(−0.640, 0.640)	.706
Final scores, median (IQR)	4 (4–4)	4 (4–4)	0.000	(−0.640, 0.640)	.758
Cases					
Reviewer 1, median (IQR)	4 (4–4)	4 (4–4)	–	–	1.000
Reviewer 2, median (IQR)	4 (4–4)	4 (4–4)	–	–	1.000
Final scores, median (IQR)	4 (4–4)	4 (4–4)	–	–	1.000

ChatGPT = Chat Generative Pre-trained Transformer, CI = confidence interval, FAQs = frequently asked questions, IQR = interquartile range.

### 3.2. Performance of DeepSeek V3 and ChatGPT-4o in 2 runs on esophageal cancer-related questions

In both runs, DeepSeek V3 and ChatGPT-4o showed favorable temporal stability for responses to questions related to esophageal cancer, with no statistically significant differences in the overall scores as well as the scores of each subgroup (Table S3, Supplemental Digital Content 3, Figs. 2 and 3). Specifically, DeepSeek V3 produced inconsistent responses on 2 questions, while ChatGPT-4o produced different responses on only 1 question between the 2 runs. DeepSeek V3 and ChatGPT-4o performed well in responding to basic knowledge-related questions and could provide comprehensive responses. In the first run, DeepSeek V3 was able to provide accurate answers to almost all questions, although some responses were considered incomplete. ChatGPT-4o was evaluated with a score of 2 for 1 response each in diagnosis and molecular biology, management of local and locoregional diseases, and management of advanced and metastatic diseases, respectively (Table 2).

**Table 2:** Scores of DeepSeek V3 and ChatGPT-4o on different sections of esophageal cancer related questions.

DeepSeek V3	1 point	2 points	3 points	4 points
Basic knowledge, n (%)	0	0	4 (30.8)	9 (69.2)
Diagnosis and molecular biology, n (%)	0	0	1 (33.3)	2 (66.7)
Management of local and locoregional diseases, n (%)	0	0	2 (40.0)	3 (60.0)
Management of advanced and metastatic diseases, n (%)	0	0	5 (62.5)	3 (37.5)
Case	0	0	0	3 (100%)
FAQs	0	1 (5.0)	2 (10.0)	17 (85.0)
ChatGPT-4o				
Basic knowledge, n (%)	0	0	4 (30.8)	9 (69.2)
Diagnosis and molecular biology, n (%)	0	1 (33.3)	1 (33.3)	1 (33.3)
Management of local and locoregional diseases, n (%)	0	1 (20.0)	1 (20.0)	3 (60.0)
Management of advanced and metastatic diseases, n (%)	0	1 (12.5)	4 (50.0)	3 (37.5)
Case	0	0	0	3 (100%)
FAQs	0	0	4 (20.0)	16 (80.0)

ChatGPT = Chat Generative Pre-trained Transformer, FAQs = frequently asked questions.

**Figure 2.**
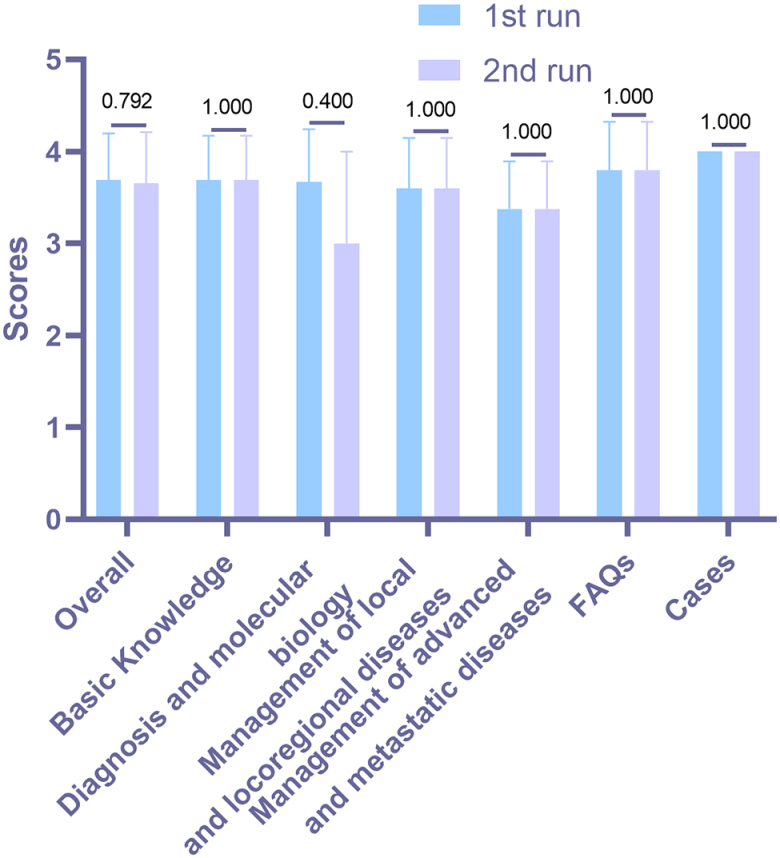
The overall scores and different subdomains of DeepSeek V3 for answering esophageal cancer-related questions in 2 runs. FAQ = frequently asked question.

**Figure 3.**
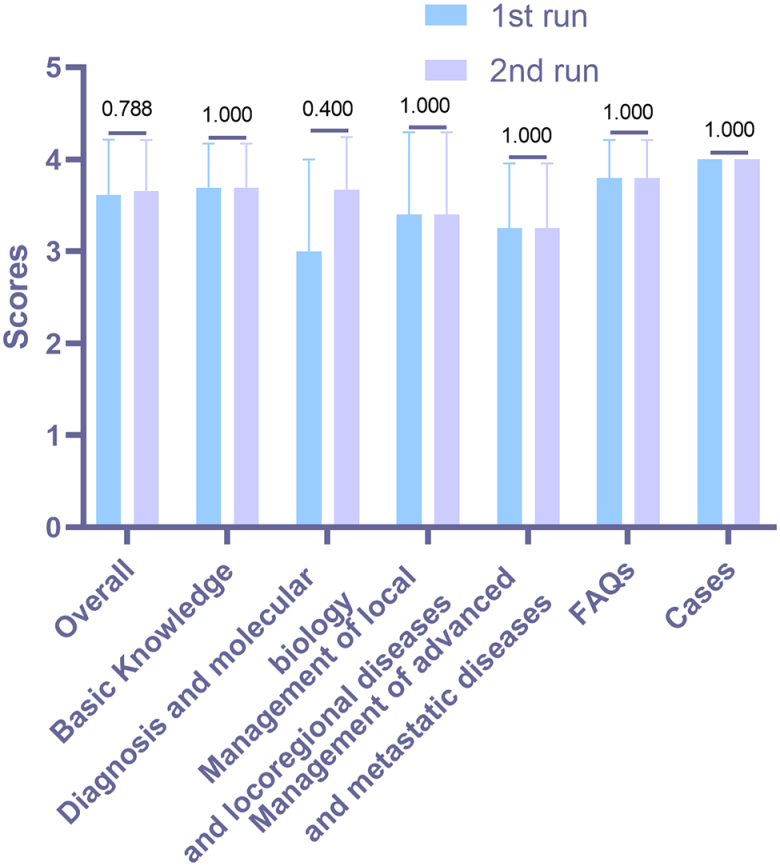
The overall scores and different subdomains of ChatGPT-4o for answering esophageal cancer-related questions in 2 runs. ChatGPT = Chat Generative Pre-trained Transformer, FAQ = frequently asked question.

### 3.3. Error types of DeepSeek V3 and ChatGPT-4o responses to esophageal cancer-related questions

Table 3 showed the error types of DeepSeek V3 and ChatGPT-4o responses to esophageal cancer-related questions. The error types of model responses were classified into 3 mutually exclusive categories: omissions, outdated information, and factual inaccuracies. Each individual response was assigned to only 1 category based on the most prominent error observed, ensuring that a single answer could not be counted in more than 1 category. Omission was the most common error type in DeepSeek V3 and ChatGPT-4o, with 28.8% and 26.9% of the questions answered correctly but insufficiently. ChatGPT-4o gave outdated information in 2 of the questions. In addition, both DeepSeek V3 and ChatGPT-4o ‘s responses to one of the questions included factually incorrect answers. In the second run, DeepSeek V3 failed to correctly answer the question “For esophageal cancer, diagnosis should be made by histopathological evaluation of a minimum number of endoscopic biopsies to ensure adequate representation of the tumor and sufficient tissue for molecular analysis?.” ChatGPT-4o failed to produce a correct response to the question on the first run, but answered it correctly on the second run.

**Table 3:** Error types of DeepSeek V3 and ChatGPT-4o responses to esophageal cancer related questions.

	DeepSeek V3 (n = 52)		ChatGPT-4o (n = 52)	
	First run	Second run	First run	Second run
Omissions, n (%)	15 (28.8)	15 (28.8)	14 (26.9)	14 (26.9)
Outdated information, n (%)	0	0	2 (3.8)	2 (3.8)
Factual inaccuracies, n (%)	0	1 (1.9)	1 (1.9)	0

ChatGPT = Chat Generative Pre-trained Transformer.

## 4. Discussion

Esophageal cancer is a common digestive tract tumor, and its early stage usually has no obvious symptoms.^[29]^ Due to the lack of understanding of health knowledge related to esophageal cancer, many patients have progressed to advanced stages at the time of diagnosis. It has been reported that some patients attribute difficulty in swallowing to normal or incorrect physical reactions, believing that they “swallow food in the wrong way” or “do not chew food correctly,” and thus do not realize the reason for seeking medical help.^[30,31]^ The development of Internet technology has provided the public with quick and convenient access to health knowledge. However, a study evaluated the quality of online information for esophageal cancer and found that few websites provided accurate incidence/prevalence, staging, prognosis, or prevention information, and the quality of websites providing information on esophageal cancer was variable.^[32]^ Unlike search engines, artificial intelligence chatbots (such as ChatGPT-4o) provide interactive, generative responses and understand context, making them more similar to human conversation, providing a peer-to-peer reply to searchers.^[33]^ However, the performance of DeepSeek V3 and ChatGPT-4o in answering esophageal cancer-related questions has not been evaluated.

In this research, we found that the scores of DeepSeek V3 and ChatGPT-4o on all questions were 4 (3–4), which indicated that DeepSeek V3 and ChatGPT-4o could provide relatively comprehensive responses when answering esophageal cancer-related health questions. This indicates that DeepSeek V3 and ChatGPT-4o exhibited favorable overall accuracy and temporal stability in responding to esophageal cancer-related public health knowledge and clinical queries. However, the current evidence is insufficient to support their use as fully reliable clinical or patient tools. Instead, these models may hold potential as auxiliary informational aids to supplement patient education and clinical reference, but they cannot replace professional medical advice, oncological expertise, or formal clinical decision-making.

For FAQs, both models achieved high scores, indicating their ability to provide accurate and comprehensive responses to common patient concerns. However, for the 3 clinical case analysis questions, which required integrated reasoning and application of guideline knowledge, DeepSeek V3 and ChatGPT-4o scored 4 (4–4). A study evaluated the accuracy and temporal stability of ChatGPT-4o in addressing Helicobacter pylori-related issues, and the results show that it can provide correct answers for most Helicobacter pylori-related queries.^[27]^ However, DeepSeek V3 and ChatGPT-4o also sometimes provide wrong answers. For example, when asked, “For esophageal cancer, diagnosis should be made by histopathological evaluation of a minimum number of endoscopic biopsies to ensure adequate representation of the tumor and sufficient tissue for molecular analysis?,” ChatGPT-4o incorrectly answered “a minimum of 4 to 6 biopsies is typically recommended” in one of the runs, while DeepSeek V3 also incorrectly answered in 1 of the runs. Currently, guidelines usually recommend at least 6 to 8 biopsies. For primary care physicians lacking sufficient relevant knowledge, obtaining incorrect answers may lead to missed diagnoses and delay the treatment of patients. In addition, we found no significant difference in response scores between DeepSeek V3 and ChatGPT-4o in the 2 runs, indicating that both models demonstrated good temporal stability across the 2 independent evaluation runs.

For patients, LLMs serve primarily as educational tools to address general questions, reduce information asymmetry, and improve health literacy; here, risks center on oversimplification, misinterpretation, or incomplete guidance. In contrast, clinician-facing use involves high-stakes tasks such as guideline consultation and treatment-related reasoning, where even minor inaccuracies may compromise clinical decision-making and patient safety – hence ESMO’s explicit caution against unsupervised use in oncology. Our findings show both models deliver generally accurate esophageal cancer-related information, supporting cautious patient-facing use under professional supervision. However, their moderate consistency, non-validated scoring, and lack of harm assessment preclude standalone clinician-facing application. Rigorous oversight, multidisciplinary validation, and adherence to ESMO recommendations remain essential for safe, responsible use across both contexts. Esophageal cancer-specific LLM research has expanded, yet critical gaps persist. Recent work largely focuses on single-model assessments (e.g., ChatGPT-4o) for narrow tasks like patient education or radiological staging, lacking comprehensive multi-model comparisons across diverse clinical scenarios. No study has systematically evaluated both DeepSeek V3 and ChatGPT-4o using a curated 52-question set spanning basic knowledge, FAQs, guideline adherence, and clinical reasoning. Additionally, existing literature overlooks temporal stability – key for public LLMs with unannounced backend updates – and rarely complies with 2025 standardized reporting guidelines.

Despite the favorable overall performance observed, the clinical interpretability and generalizability of our findings are constrained by several core limitations that should guide all result interpretations. Statistically, the small sample sizes of certain subgroups (e.g., n = 3 for diagnosis and molecular biology; n = 5 for local/locoregional management) render p‑values unstable and comparisons underpowered. We therefore emphasize effect sizes and confidence intervals over p‑values, and acknowledge that all subgroup analyses are exploratory and hypothesis‑generating rather than confirmatory. No formal adjustments for multiple comparisons can fully overcome the limited statistical power inherent in these small subgroups. First, this study evaluated only 52 manually selected esophageal cancer-related questions and just 3 clinical cases, representing a narrow, nonrepresentative sample that limits extrapolation to diverse real-world patient or clinical inquiries. Second, we employed a non-validated composite ordinal score integrating accuracy, completeness, and clinical risk without formal calibration, which may affect scoring objectivity and comparability. Third, several clinically critical dimensions were not assessed, including potential harmfulness of outputs, evidence-based citation support, patient-facing readability, and clinical actionability – all essential for safe LLM deployment in oncology. Additionally, inherent limitations of current LLMs must be acknowledged: their responses synthesize vast information into accessible language, yet regional variations in expert guidance may exist, and individuals without medical backgrounds may struggle to identify reliable information. Moreover, model outputs lack explicit references or source attribution, limiting traceability and trustworthiness. Critically, the expert review panel comprised only gastroenterologists, without input from medical oncologists, thoracic surgeons, or pathologists, which may introduce specialty-specific bias. Future work should expand clinical case numbers and adopt a multidisciplinary review panel to enable more comprehensive, subspecialty-informed evaluation. Finally, the 2 independent tests assessed temporal stability rather than strict temporal stability. Public LLMs may undergo unannounced backend updates or fine-tuning during the 1-week interval; thus, our findings reflect real-world consistency over time – a distinction critical for interpreting LLM performance in routine public use. Collectively, these cumulative limitations mandate an extremely cautious interpretation: our results represent only preliminary evidence of informational potential, not validation for unassisted clinical or patient use.

In conclusion, DeepSeek V3 and ChatGPT-4o achieved high scores and generated generally comprehensive responses on the specific set of 52 esophageal cancer-related questions evaluated in this study. Nevertheless, both models produced omissions, outdated information, and occasional factual inaccuracies. These findings indicate favorable performance within this benchmark but should be interpreted only in the context of the evaluated question set and should not be considered evidence of general reliability for esophageal cancer-related clinical or public use.

## Author contributions

**Conceptualization:** Qian Yang, Yunkai Zhan.

**Data curation:** Qian Yang, Jing Yi, Anran Gong, Aiyan Fu, Yunkai Zhan.

**Formal analysis:** Qian Yang, Jing Yi, Jiangtao Li.

**Funding acquisition:** Jing Yi, Jiangtao Li.

**Investigation:** Qian Yang, Anran Gong, Qingyan Feng.

**Methodology:** Anran Gong, Aiyan Fu.

**Project administration:** Yanyan Li, Aiyan Fu, Yunkai Zhan.

**Resources:** Yanyan Li, Yunkai Zhan.

**Software:** Jing Yi, Qingyan Feng.

**Supervision:** Yanyan Li, Yunkai Zhan.

**Validation:** Qingyan Feng, Jiangtao Li.

**Writing – original draft:** Qian Yang, Jing Yi.

**Writing – review & editing:** Yunkai Zhan.






