# Supplementary material for: Performance of DeepSeek V3 and ChatGPT-4o in answering esophageal cancer-related questions
Source: Medicine (Baltimore). 2026 Jul 24;105(30):e49896. doi: 10.1097/MD.0000000000049896 (PMC13406192; doi:10.1097/MD.0000000000049896)
Supplement: Supplementary file 1 [file medi-105-e49896-s001.docx]

**Supplementary Table S1.** Standardized inference protocol

| **Item** | **Description** |
| --- | --- |
| Model | DeepSeek V3 / ChatGPT-4o |
| Access | Public web interface |
| Prompt | Original question only |
| Prompt engineering | None |
| System prompt | Default platform setting |
| Input format | Verbatim question |
| Conversation | New chat for every question |
| Follow-up prompts | None |
| Regeneration | None |
| Manual editing | None |
| Temperature | Default (not user configurable) |
| Top-p | Default (not user configurable) |
| Max output length | Default |
| Memory | Disabled |
| Browsing history | Disabled |
| Test dates | April 11 and April 18, 2025 |
