# Supplementary material for: Performance of DeepSeek V3 and ChatGPT-4o in answering esophageal cancer-related questions
Source: Medicine (Baltimore). 2026 Jul 24;105(30):e49896. doi: 10.1097/MD.0000000000049896 (PMC13406192; doi:10.1097/MD.0000000000049896)
Supplement: Supplementary file 3 [file medi-105-e49896-s003.docx]

| Supplementary Table 3. Performance of DeepSeek V3 and ChatGPT-4o on esophageal cancer related questions in different runs. | | | | | |
| --- | --- | --- | --- | --- | --- |
|  | 1st run | 2nd run | Effect size | 95% CI | p value |
| Overall |  |  |  |  |  |
| DeepSeek V3, median (IQR) | 4 (3-4) | 4 (3-4) | 0.072 | (-0.317, 0.461) | 0.788 |
| ChatGPT-4o, median (IQR) | 4 (3-4) | 4 (3-4) | -0.066 | (-0.455, 0.323) | 0.792 |
| Basic Knowledge |  |  |  |  |  |
| DeepSeek V3, median (IQR) | 4 (3-4) | 4 (3-4) | 0.000 | (-0.809, 0.809) | 1.000 |
| ChatGPT-4o, median (IQR) | 4 (3-4) | 4 (3-4) | 0.000 | (-0.809, 0.809) | 1.000 |
| Diagnosis and molecular biology |  |  |  |  |  |
| DeepSeek V3, median (IQR) | 4 (3-4) | 3 (2-4) | 0.816 | (-1.544, 3.176) | 0.400 |
| ChatGPT-4o, median (IQR) | 3 (2-4) | 4 (3-4) | -0.816 | (-3.176, 1.544) | 0.400 |
| Management of local and locoregional diseases |  |  |  |  |  |
| DeepSeek V3, median (IQR) | 4 (3-4) | 4 (3-4) | 0.000 | (-1.459, 1.459) | 1.000 |
| ChatGPT-4o, median (IQR) | 4 (2.5-4) | 4 (2.5-4) | 0.000 | (-1.459, 1.459) | 1.000 |
| Management of advanced and metastatic diseases |  |  |  |  |  |
| DeepSeek V3, median (IQR) | 3 (3-4) | 3 (3-4) | 0.000 | (-1.073, 1.073) | 1.000 |
| ChatGPT-4o, median (IQR) | 3 (3-4) | 3 (3-4) | 0.000 | (-1.073, 1.073) | 1.000 |
| FAQs |  |  |  |  |  |
| DeepSeek V3, median (IQR) | 4 (4-4) | 4 (4-4) | 0.000 | (-0.640, 0.640) | 1.000 |
| ChatGPT-4o, median (IQR) | 4 (4-4) | 4 (4-4) | 0.000 | (-0.640, 0.640) | 1.000 |
| Cases |  |  |  |  |  |
| DeepSeek V3, median (IQR) | 4 (4-4) | 4 (4-4) | - | - | 1.000 |
| ChatGPT-4o, median (IQR) | 4 (4-4) | 4 (4-4) | - | - | 1.000 |
